# Improving and preserving cardiorespiratory fitness, muscle strength and adiposity through a complex lifestyle intervention in community-dwelling older adults with elevated cardiometabolic risk: study protocol for the RESTART randomised controlled trial

**DOI:** 10.1136/bmjopen-2024-095810

**Published:** 2025-04-19

**Authors:** Jonas Johansson, Trygve Sigvart Deraas, Laila Arnesdatter Hopstock, André Henriksen, Sameline Grimsgaard

**Affiliations:** 1 Department of Community Medicine, UiT The Arctic University of Norway, Tromso, Norway; 2 Department of Health and Care Sciences, UiT The Arctic University of Norway, Tromsø, Norway; 3 Department of Computer Science, UiT The Arctic University of Norway, Tromso, Norway

**Keywords:** Randomized Controlled Trial, Aging, Exercise, Behavior, NUTRITION & DIETETICS, Primary Prevention

## Abstract

**Introduction:**

As the global population ages, the incidence of cardiometabolic diseases and associated healthcare costs rise. There is a critical need for preventive interventions enabling long-lasting treatment effects to address the decline in physical performance and metabolic health among older adults. The RESTART (RE-inventing Strategies for healthy Ageing: Recommendations and Tools) randomised controlled trial (RCT) aims to evaluate whether a complex lifestyle intervention can improve and maintain cardiorespiratory fitness, muscle strength and body composition among older adults with elevated cardiometabolic risk.

**Methods and analysis:**

This is the study protocol for the RESTART trial, a two-arm, open-label, parallel-group RCT conducted in Tromsø, Norway, targeting adults aged 60–75 with obesity, a sedentary lifestyle and high cardiovascular risk. Participants are block-randomised (1:1) into either an intervention or active control group. The initial intervention phase (12 months) includes: (a) supervised high-intensity aerobic and strength training (≥85% of maximum capacity) performed two times weekly, (b) behavioural counselling based on acceptance and commitment therapy during six group sessions and (c) dietary guidance based on national nutrition recommendations during two group/two individual sessions. After 12 months, participants are gradually introduced to exercise sessions offered by local organisations and fitness centres, to enable independent maintenance of lifestyle change. The primary outcome is a change in cardiorespiratory fitness (V̇O_2max_) at 24 months. Secondary and tertiary outcomes include additional parameters potentially sensitive to lifestyle change, such as 1-repetition maximum muscle strength, muscular power, device-measured physical activity levels, body composition, waist circumference, body weight, cognitive function and self-reported health-related quality of life. Data collection is scheduled at baseline, 6, 12 and 24 months, with health economic and qualitative analyses to evaluate the intervention’s impact and participant experiences.

**Ethics and dissemination:**

Ethical approval for the RESTART trial was obtained from the Regional Committee for Medical Research Ethics in Northern Norway. Results will be disseminated through peer-reviewed publications, conferences and community-based channels targeting older adults, healthcare providers and municipal health organisations. This trial will also inform public health strategies for lifestyle interventions among ageing populations.

**Trial registration number:**

NCT06122441.

**STRENGTHS AND LIMITATIONS OF THIS STUDY**

- This complex randomised controlled trial (RCT) will assess the combined health impact of three key lifestyle interventions: physical training, behavioural strategies and dietary counselling.
- A long follow-up period (24 months) allows for evaluation of the durability of intervention effects.
- The study will incorporate a range of health outcomes across multiple domains, such as aerobic capacity, muscle strength, adiposity, quality of life, cognitive function, self-efficacy, self-compassion and dietary behaviours.
- The target population is clinically relevant, focusing on older adults with obesity, cardiometabolic risk and physical inactivity.
- The RCT is not statistically powered to detect significant changes in hard endpoints, such as myocardial infarction or mortality.

## Introduction

### Background and rationale

 Globally, the proportions of older adults are increasing, with a doubling of individuals over the age of 65 in 2050.^1^ Unless effective preventive actions are implemented, we face an increase in disease burden and healthcare expenditure, as ageing is associated with increased incidence of cardiometabolic diseases such as myocardial infarction, stroke and type 2 diabetes,^2 3^ and states of reduced physical function such as frailty and sarcopenia.^4 5^ The progression of such conditions is likely driven in part by reduced physical activity among older adults, which would contribute to observed age-related reductions in aerobic capacity (V̇O_2max_), muscle strength and physical performance.^6^,^8^ Less physical activity coupled with reduced basal metabolic rate in older age, without any proportional reduction in energy intake, also contributes to increased abdominal adiposity,^9^ which is central in cardiometabolic disease progression.^10^

Primary prevention efforts could beneficially target several lifestyle factors to meet the health challenges of the ageing population. Aerobic endurance and strength training of high intensity (≥85% of maximal capacity) have shown to be well tolerated and efficiently improve V̇O_2max_ and muscle strength respectively in older adults, physical capacities that are strongly related to cardiovascular disease (CVD), mortality and fracture incidence.^11^,^15^ Lifestyle interventions that combine physical exercise with behavioural and/or dietary counselling are additively effective compared with focusing on each lifestyle behaviour separately.^16 17^ Such simultaneous targeting of several interacting elements is the hallmark of complex intervention design,^18^ which can be used to address the multifaceted real-life settings in which lifestyle change often occurs.

However, lifestyle interventions often fail to demonstrate long-term maintenance of intervention effects. Comprehensive evidence shows that nearly 50% of participants undergoing behavioural treatment for obesity may regain their baseline weight within 5 years,^19^ and that patients with diabetes partaking in an extensive lifestyle programme may return their physical activity and diet behaviours to initial levels within 1–5 years of post-treatment.^20^ Similarly, the multi-centre Look AHEAD trial noticed a marked recoil effect in waist circumference, physical fitness and metabolic markers in the years following participation in a lifestyle intervention.^21^ As pointed out by a recent meta-analysis, future trials need to focus on long-term interventions to properly establish and evaluate changes in lifestyle.^22^ Others have proposed that successful maintenance of lifestyle change may require multisectoral cooperation between researchers, municipalities, non-governmental organisations (NGOs) and the health-fitness industry to improve health in vulnerable, older populations.^23^ An increased focus on the retirement phase to facilitate establishing new healthy lifestyle habits among older adults has also been suggested,^24^ especially if aided by primary prevention models gradually supporting people’s self-management of health.^25^ Collectively, the knowledge gaps and potential approaches identified in these studies highlight the need to investigate complex, multisectoral and long-term prevention models in order to achieve lasting lifestyle change in vulnerable older adults with cardiometabolic risk.

We have previously evaluated the feasibility of the RESTART (RE-inventing Strategies for healthy Ageing: Recommendations and Tools) pilot study, a 6-month complex lifestyle intervention occurring between 2017 and 2018 (clinicaltrials.gov NCT03807323).^26^ We recruited 16 participants aged 57–74 years with obesity, a sedentary lifestyle and elevated CVD risk from the seventh survey of the Tromsø Study (Tromsø7; 2015–2016). Participants underwent a group-supervised, high-intensity aerobic endurance and strength training programme including behavioural and dietary counselling, with 71% attendance and without dropouts. Semi-structured, individual and focus group interviews reported the intervention as feasible and well-tolerated by the participants.^26^

The feasibility results from the pilot study supported proceeding to a full-scale randomised controlled trial (RCT) to evaluate how a complex lifestyle intervention can support participants in gradually and independently maintaining an improved lifestyle. The Research Council Norway (RCN) granted funding from 2023, and the study begins participant enrolment in January 2024. This paper outlines the study protocol for the RESTART trial.

### Objectives

The RESTART trial aims to examine whether a long-term complex lifestyle intervention involving supervised physical training, dietary and behavioural counselling, and coordinated with municipal and NGOs, can establish lasting improvements in lifestyle, functional capacity and risk factors in older adults at high risk of cardiometabolic disease.

#### Primary objectives

We hypothesise that, compared with an active control group, the complex intervention will at 24 months have:

1. Produced a clinically relevant increase in cardiorespiratory fitness (primary endpoint).
2. Increased muscle strength, physical activity level and reduced adiposity
3. Improved body composition, health-related quality of life and cognitive function.

#### Secondary objectives

The RESTART trial will additionally:

1. Explore participants’ experiences and key determinants of lasting lifestyle change in older adults.
2. Evaluate health economic implications of the intervention.

### Trial design

The RESTART trial is designed as a two-arm, open-label parallel group RCT, with a block-randomised 1:1 allocation ratio into either intervention or active control group. Both groups will be assessed at baseline, 6, 12 and 24 months, with the primary endpoint cardiorespiratory fitness assessed at 24 months. The RESTART trial will test the hypothesised superiority of a supervised complex lifestyle intervention against simple monitoring of physical activity and general lifestyle advice according to national recommendations.

## Methods: participants, interventions, and outcomes

### Study setting

The RESTART trial conduct and data collection will take place in Tromsø, Norway (pop. 79000) from 2023 to 2026. Pre-identification of potential participants will be performed (starting in September 2023) using data collected in Tromsø7, 2015–2016. The Tromsø Study was initiated in 1974 to study risk factors, causes and prevention of CVD, and is Norway’s most longstanding and comprehensive population study. Throughout seven consecutive surveys, it has expanded to cover a wide range of risk factors, chronic diseases, use of healthcare services and disease endpoints.^27^

Screening and initial assessment of inclusion/exclusion criteria will be performed (November 2023) by the RESTART trial coordinator at the Department of Community Medicine, UiT The Arctic University of Norway (UiT). The Clinical Trial Unit (CTU) at the local university hospital will perform additional screening through clinical examinations and blood and urine sampling and additionally assess body composition and physical function. Testing of participants’ V̇O_2max_, muscular strength and power will take place at the UiT Faculty of Health Sciences research laboratory for sports, physical activity and public health. Study participants are subsequently enrolled (January 2024) via randomisation to either intervention or control. The intervention participants will undertake aerobic endurance and strength training at KRAFT sports centre at the UiT campus area. This exercise centre also offers rooms for dietary and behavioural counselling activities.

### Eligibility criteria

All participants are asked to provide written, informed consent before proceeding in the trial (see online supplemental file 1). As the study’s aim is to include a study population of older adults at high risk of cardiometabolic disease, the following inclusion criteria will be applied:

1. 60–75 years of age at the year of inclusion.
2. Body mass index (BMI) ≥30 kg/m^2^.
3. Elevated CVD risk, defined as scoring 8/12 on the NORRISK 2 model.^28^
4. Sedentary lifestyle, defined as the lowest level on the Saltin-Grimby Physical Activity Level Scale.^29^

Inclusion criteria #1 is applied to ensure the inclusion of a segment of the older adult population that may still retain sufficient physical function to be able to attend a comprehensive intervention programme containing high-intensity exercise. Inclusion criteria #2–4 are applied to ensure a study population with established risk factors for cardiometabolic disease.

Participants are excluded based on the presence of any of the following diagnoses or conditions, ascertained during telephone screening and baseline assessments:

1. Presence of dementia diagnosis.
2. Self-reported previous myocardial infarction or stroke, and/or self-reported established CVD by the presence of coronary stent.
3. Heart failure with ejection fraction <50%.
4. ECG presenting with atrioventricular block grade 2 type 2 or grade 3, or previous myocardial infarction.
5. Uncontrolled hypertension (systolic blood pressure ≥180 mm Hg and/or diastolic blood pressure ≥110 mm Hg unless cleared by a nephrologist).
6. Chronic obstructive pulmonary disease grade 3 or 4.
7. Thyroid dysfunction.
8. Liver dysfunction.
9. Kidney dysfunction.
10. Severe anaemia.
11. Uncontrolled diabetes.
12. Severe hearing problems.
13. Severe mobility limitations (unable to get up from a sitting or lying position, unable to maintain a crouched position, unable to raise arms above shoulder or head height).
14. No possession of a smartphone.
15. No access to the Norwegian national identification service (BankID).
16. Unable to follow the trial regimen for 24 months.

Exclusion criteria 7–11 are based on the analysis of blood samples measured at baseline, with cut-off values listed as online supplemental file 2. The general rationale for applying the above-listed exclusion criteria is to ensure that participants are physically able and medically approved to receive a complex intervention programme, which includes managing the physical demands of high-intensity training, besides receiving the health benefits.

### Interventions

Before randomisation, the intervention and control groups will be given a wrist-worn activity tracker to measure physical activity and energy expenditure throughout the trial. Participants randomised to the control group will also receive a brief counselling and a written hand-out based on Norwegian exercise and nutritional guidelines to promote healthy lifestyle habits.

The intervention group progresses through a complex intervention comprising high-intensity physical training, behavioural counselling based on acceptance and commitment theory (ACT)^30^ and dietary counselling based on Norwegian nutritional guidelines. Drawing on feasibility study experiences, the different intervention elements are introduced stepwise to reduce initial overload for the participants. Participants are progressively transitioned to the municipal Healthy Life Centre (HLC; in Norwegian: ‘Frisklivssentralen’) and further to local NGOs, who deliver activities harmonised with the intervention elements. They will be supported by a web-based eTool which digitally reinforces the intervention. Overall, the intervention is designed to progressively enable participants to make independent and personal healthy lifestyle choices, and to use the skills from the physical training, ACT and diet intervention elements attained in the initial 12 months.

#### Supervised high-intensity endurance and strength training (element A)

Physical training is well-documented as a strategy to improve V̇O_2max_ and maximal muscle strength in older adults, with improvements similar to what is observed in younger populations.^12 13^ The risk of adverse effects is minimal if executed correctly. Intervention participants will be organised into four groups of 13–15 participants each and perform high-intensity physical training guided by a certified training instructor, to target the cardiovascular system and skeletal muscle force-generating capacity aiming for optimal outcome.^12^ The intervention group will perform supervised indoor cycling twice weekly with a relatively high intensity (85%–90% of maximal heart rate (HR) during 4×4 min) separated by 3 min active rest periods at ~70% of maximal HR. HR will be continuously monitored using a physical activity tracker and chest-worn HR belt to ensure that the targeted intensity is met. This part of the training session aims to improve participants’ V̇O_2max_. Approximately 10 min after the aerobic endurance training session, the intervention group will perform horizontal leg press, chest press, and lateral pulldown or sitting row exercises (three sets of five repetitions; 3 min rest between sets) with an intensity corresponding to 85%–90% of their maximal strength, to improve skeletal muscle force generating capacity and physical function. The participants will be instructed to perform these machine-based, indoor strength training exercises with maximal intended velocity in the contraction phase for optimal neuromuscular activation. Endurance training progression is ensured by continuously encouraging participants to meet the target intensity during intervals, which will result in an increased absolute workload because of improvements in V̇O_2max_. Strength training progression is monitored by the training instructor who records the load each session and motivates participants to increase the load when they successfully complete the target sets and repetitions. The RESTART training instructor will also continuously encourage the participants to be physically active and reduce sedentary time on non-training days.

During months 6–12, one of the two weekly training sessions will continue to be led by the RESTART instructor, while the other session will be led by the HLC, enabling transition to supervised training via the Tromsø Municipality primary healthcare. The HLC will implement a primary prevention programme based on the RESTART trial training principles and primarily perform the interval-based endurance training outdoor by using terrain and natural inclines. Additionally, the strength training programme will be expanded by alternating between functional exercises (squats, deadlifts, lunges, assisted push-ups and bent-over rows) with free weights every other week and continuing with the machine-based exercises from the initial 6 months during the alternate weeks. The functional exercises are progressed through three increasingly challenging stages throughout the trial and the instructor motivates the use of heavier weights if participants effortlessly complete the sets and repetitions. From months 12 to 18, the supervised RESTART training sessions end, but the HLC will maintain one training session per week and focus on functional exercises for the strength training session. All indoor training activities delivered by the RESTART instructor and the HLC instructors occur at the Kraft sports centre and are individualised, as the exercises are adjusted and progressed based on individual physical form improvements, and/or potential pain and movement restrictions. At the 12- to 18-month phase, participants are invited to join a weekly training session organised by the non-profit National Association for Heart and Lung Disease (LHL), engage in activities offered by the Norwegian Trekking Association (DNT), or visit local fitness centres. These organisations receive education and training to support aligning their activities with the RESTART trial training principles.

#### Behavioural counselling (element B)

Between months 3 and 12, intervention participants receive behavioural counselling based on the ACT approach during six 2-hour group sessions,^30^ delivered by clinical psychologists, using the same four-group structure established for the physical training sessions. ACT can be implemented across a range of therapeutic settings, uses concepts of mindfulness and acceptance to identify person-centred values and goals in life, and is proven effective among older adults.^31^ This personalised approach delivered in a group setting aims to strengthen participants’ ability to cope with difficult cognitive and emotional experiences, and foster psychological flexibility/distress tolerance skills needed to develop self-efficacy and new behaviour patterns consistent with their life values.^32^ This is especially important to foster distress tolerance during high-intensity interval training and to develop healthier eating habits.

#### Dietary counselling (element C)

Between months 6 and 12, the intervention participants receive four dietary counselling sessions based on Norwegian dietary guidelines,^33^ and delivered by a clinical nutritionist. The goal is to introduce a healthy, sustainable and personalised diet, and reduce the intake of high-energy-dense foods and drinks with low nutritional quality. Two sessions include individual counselling constituted by a comprehensive mapping of the participant’s current diet and identifying potential actions for a healthier diet change. The two other sessions are group-based and use the same group structure from the physical training and ACT intervention sessions, focusing on basic nutritional advice delivered as a group lecture and practical food preparation together with a professional chef. For these group sessions, participants are encouraged to bring their partners, and the nutritionist is additionally familiar with the ACT principles to enhance participants’ uptake of the dietary guidelines.

#### eTool support for continued healthy lifestyle promotion (element D)

The intervention group will, from month 12, have access to the RESTART eTool, a web-based portal that is iteratively being developed by the RESTART management team together with external exercise providers, participant representatives and health professionals. It focuses on the promotion of physical activity and exercise principles, healthy dietary habits, and behavioural strategies among older adults, harmonised with the RESTART intervention elements. The eTool aims to provide short and straightforward articles, instruction videos, audio files and reminders for the participants as they are gradually transitioned to independently maintain the attained lifestyle habits and physical capacity levels. The eTool will additionally provide links to different external providers of exercise in the municipality, and to other national web resources promoting physical activity and diet, like the Norwegian Directorate of Health.

#### Continuous physical activity monitoring (element E)

All participants are provided with a wrist-worn consumer-based physical activity monitor (Garmin Forerunner 55, Garmin, Olathe, Kansas, USA) to continuously measure the number of steps, minutes in different activity intensities and energy expenditure throughout the trial, as well as HR during physical training sessions. We will use the mSpider experimental data collection tool, developed in the pilot study, to collect and store activity data.^34^

#### Lifestyle recommendations (element F)

Control group participants receive advice on healthy lifestyle habits using printed and digital information containing recommendations from The Norwegian Directorate of Health. These recommendations focus on being physically active at a moderate intensity for 150–300 min per week, maintaining a varied diet rich in plant-based foods, incorporating fruits, vegetables, whole grains and seafood more often than red meat, limiting processed meat and sweets, and choosing low-fat dairy products.^35^ Control group participants are verbally reminded of these recommendations in conjunction with each testing phase.

#### Discontinuation criteria

We will continuously record and follow-up on adverse events. Intervention participants will be stopped from continuing the trial if they experience major disease events or trauma during the intervention period, such as major myocardial infarction, stroke, fracture or any event that severely limits their ability to receive the intervention elements. Physical training may result in delayed-onset muscle soreness and participants will be informed that this is normal and temporary when starting a physical training regimen. Some may experience joint pain in knees or shoulders when performing exercises, and these participants will be offered alternate exercises to redistribute the physical workload. Intervention participants who decide to discontinue receiving the intervention are given the option to remain in the trial to enable follow-up data collection.

#### Adherence and fidelity

Adherence to the trial will be continuously monitored and registered by intervention instructors, and by assessors during the testing phases. The study coordinator is notified of prolonged periods of reduced attendance and follows up with the affected participants. Fidelity to the intervention plan is ensured by trial investigators periodically following up on intervention activities throughout the trial, with higher frequency in the initial 12 months. This includes being physically present to observe whether the activities are delivered according to protocol, and to provide corrective feedback and instructions to participants and the intervention personnel. To exemplify, we will observe during exercise sessions whether the participants’ HRs, as displayed by the physical activity tracker connected to chest-worn HR belts, are within the intended intensity zones (≥85% HRmax) during the endurance training intervals. After 12 months, we ask the external exercise providers to continue monitoring participant attendance and encourage them to align their activities with the RESTART training principles. Trial investigators will perform 1–2 visits to the external providers’ exercise sessions to observe and evaluate this alignment.

### Outcomes

The primary endpoint is change from baseline in mean V̇O_2max_ (mL/kg/min) at 24 months. V̇O_2max_ (also defined as aerobic capacity) is a common and robust measure of cardiorespiratory fitness strongly linked to longevity and clinically relevant for predicting cardiometabolic disease.^11 36 37^ Secondary endpoints are change from baseline in mean muscle strength (kilograms, kg) in the lower legs and upper-body, and mean peak power (watts, W) in the lower legs, at 24 months. These are vital parameters among older adults to maintain physical function, perform activities of daily living and reduce dependence.^5^ Secondary endpoints further include change from baseline in mean waist circumference (centimetres, cm) and mean body weight (kg) at 24 months, well-established clinical markers of abdominal adiposity which are sensitive to changes in physical activity and diet. We additionally assess device-measured physical activity, defined as number of steps, time spent sedentary and in different activity intensity levels, during each testing phase (change from baseline at 24 months) and continuously throughout the trial (time frame 24 months). This will enable potential detection of changes in the participants’ daily lifestyle habits in response to the complex lifestyle intervention. Tertiary endpoints are change from baseline in mean body composition (appendicular lean mass (grams, g), fat percentage (%), visceral fat (g)), health-related quality of life and cognitive function at 24 months. Assessing body composition is crucial to capturing beneficial lifestyle intervention effects such as increased lean mass and decreased fat mass, which more traditional clinical measurements such as BMI are usually less sensitive to. Assessment of health-related quality of life enables a more holistic evaluation of beneficial intervention effects beyond the physical sphere, and is a robust determinant of longevity.^38^ Finally, cognitive function is an important parameter due to its strong relationship with ageing and dementia, and its sensitivity to physical training.^39^

**Figure 1:**
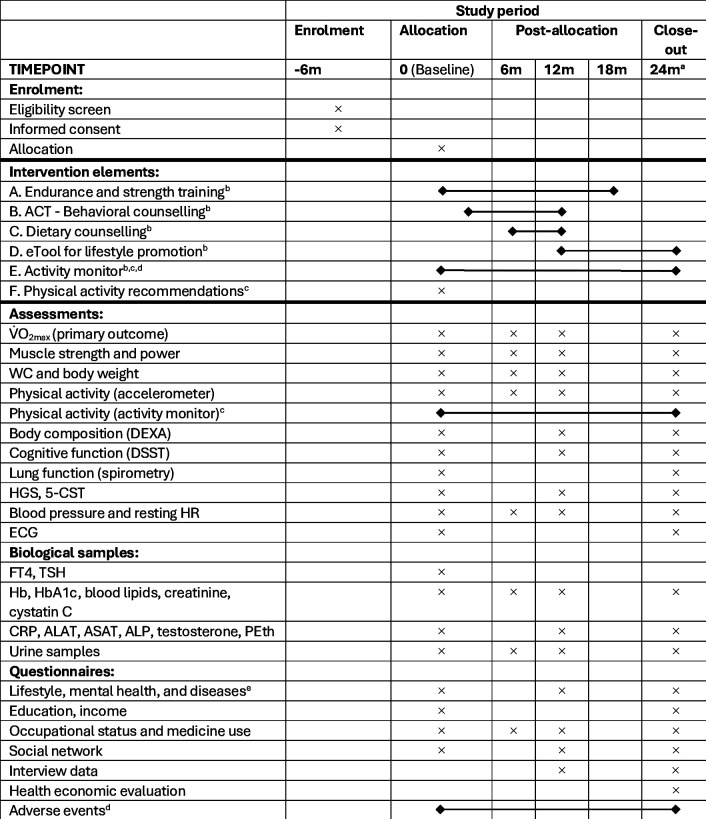
Participant timeline. ^a^Time-point for primary outcome assessment. ^b^Received by the intervention group. ^c^Received by the control group. ^d^Monitored continuously. ^e^Includes assessment of health-related quality of life (EQ-5D-5L), life satisfaction, anxiety and depression, physical activity, diet (NORKOST), general self-efficacy, self-compassion, physical activity acceptance and enjoyment, emotional and intuitive eating, binge eating episodes, ideal weight, chronic disease, symptoms and complaints, medication use, tobacco use, alcohol use and self-rated health. ACT, acceptance and commitment therapy; ALAT, alanine aminotransferase; ALP, alkaline phosphatase; ASAT, aspartate aminotransferase; CRP, C reactive protein; DEXA, dual-energy X-ray absorptiometry; DSST, Digit Symbol Substitution Test; FT4, thyroxine; Hb, haemoglobin; HbA1C, glycated haemoglobin; HGS, handgrip strength; HR, heart rate; PEth, phosphatidylethanol; TSH, thyroid-stimulating hormone; WC, waist circumference; 5-CST, 5-repetition chair stand test.

Other outcomes include change from baseline in mean resting HR (bpm), mean blood pressure (mm Hg), lung function (peak expiratory flow, L/min; forced expiratory volume, L/s; forced vital capacity, L), cardiac abnormalities using ECG, mean handgrip strength (kg) and mean 5-repetition chair stand test (5-CST) performance (seconds, s), at 24 months. Other outcomes further involve questionnaire data on perceived general self-efficacy, self-compassion, life satisfaction, mental health status, physical activity acceptance and enjoyment, emotional and intuitive eating behaviours, diet, diseases and complaints, medication use, socioeconomic status, social network, tobacco use and alcohol use at 24 months. Non-fasting venous blood samples are collected and analysed for change from baseline at 24 months (see details under the Data collection methods section and in figure 1). We additionally assess self-perceived determinants of successful lifestyle change at 24 months by semi-structured interviews. We will also perform a health economic evaluation of the intervention after 24 months, and follow-up on time to CVD events (myocardial infarction, stroke), diabetes and mortality at 60 months.

### Sample size

For the primary endpoint, we will need 44 participants in each group to detect a 2.0 mL/kg/min mean group difference in V̇O_2max_ -change, with an α-level of 0.05, power 80% and SD of 3.3. This SD is identical to the observed SD for V̇O_2max_ among pilot study participants.^40^ An effect size of 2.0 mL/kg/min is a conservative estimate that still corresponds to a 10%–15% reduction in CVD mortality risk. For secondary endpoints, sample sizes enable detection of the following between-group differences: lower body strength of 50 kg (SD=40, power>90%) and waist circumference of 4 cm (SD=3.5, power>90%). Accounting for an expected acceptance rate of 30% and an attrition rate of 20%, we aim to invite 353 individuals and randomise a total number of 110 participants into the intervention and active control groups.

## Recruitment

We have identified 365 eligible individuals who participated in Tromsø7 and fulfil the inclusion criteria based on analysed survey data. These individuals will receive written and verbal information regarding the study asking if they are willing to participate. On agreeing to participate, they will undergo a short (10–15 min) initial telephone screening to evaluate compliance with the inclusion and exclusion criteria, which is further determined during the CTU visit. If we are unable to fill the study groups, we will first promote the study in media and on webpages. Second, we will adjust the inclusion criteria of BMI≥30 kg/m^2^ to ≥28 kg/m^2^. Third, we will include participants from outside the Tromsø7 survey.

### Participants and public involvement

Participants’ experiences from the pilot study (completed in 2018) helped shape the RESTART trial study design. They suggested introducing the intervention elements stepwise to prevent initial overload, emphasised the importance of group interaction and advised that 6 months is insufficient time to establish lasting lifestyle habits. Participants from the main trial will be involved in shaping the dissemination activities and message, through group-based discussions near trial completion.

## Methods: assignment of interventions

### Allocation

#### Sequence generation

Participants will be assigned randomly to either intervention or control group with a 1:1 allocation ratio, stratified on sex, using a computer-generated randomisation schedule with permuted blocks of random sizes. To exemplify, in a block of eight participants containing an equal number of men (n=4) and women (n=4), an equal number of men (n=2) and women (n=2) will also be randomised into either the intervention or control group. The block sizes will not be disclosed to outcome assessors or data analysts running the main analyses, to ensure concealment.

#### Allocation concealment mechanism

The randomisation will be performed using the Research Electronic Data Capture (REDCap) web platform (V.13.10.4, Vanderbilt University, Nashville, Tennessee, USA), which is the primary data collection software service used in the trial by the study coordinator and the CTU. The allocation mechanism is built into the REDCap software and is unknown to either the study personnel performing the randomisation or the participants. Allocation results are stored securely on the software server inaccessible by outcome assessors and remain concealed for them until after the participants have completed the trial. The randomisation code is released after study completion.

#### Implementation

All eligible participants who give their consent will be randomised into the trial after they have completed all baseline assessments during the visits to the CTU and the research laboratory for sports, physical activity and public health. The REDCap software automatically generates the randomisation sequence from pre-specified criteria such as sex-stratification and assigns participants to intervention or control groups. Participants are subsequently enrolled into the trial by the study coordinator who further informs the study management team and responsible intervention instructors.

### Blinding (masking)

Outcome assessors will be blinded to group assignments from baseline to 24-month follow-up. It is not possible to blind the participants or intervention staff due to the nature of the intervention. Participants and intervention staff will be given explicit instructions to not reveal the group allocation to the outcome assessors during each testing phase, in order to maintain the blinding.

## Methods: data collection, management and analysis

### Data collection methods

#### Primary outcome

V̇O_2max_ will be assessed during an incremental test-to-exhaustion where the participant walks or runs on a motorised treadmill (Lode Katana Sport XL, Lode, Groningen, Netherlands). V̇O_2max_ data will be obtained from the participant via a face mask connected to a Vyntus CPX portable ergospirometry system (Vyaire Medical, Mettawa, Illinois, USA) which will be set in mixing chamber mode and is worn on the participant’s back. The participant will additionally wear an HR sensor chest belt connected to the ergospirometry software for continuous HR tracking during the test. Baseline measurements will include a 12-point ECG test ahead of the V̇O_2max_ assessment to medically clear the participant for testing. Participants with a systolic blood pressure between 160 and 179 mm Hg are temporarily halted from testing until their blood pressure is under control (<160 mm Hg).

Test instructors will initiate the warm-up session by having the participant walk for 5 min on the treadmill using slow velocity and no incline for familiarisation purposes. Participants will then receive the face mask and ergospirometry system and walk for five more minutes where the test instructor subjectively assesses the participant’s respiratory exchange ratio (RER). This concludes the warm-up session, and the test instructor will initialise the test with a velocity between 3–5 km/hour and 0%–4% incline based on the RER values during warm-up. Thereafter, only the treadmill inclination is increased, by 2% every 2 min until exhaustion. Participants will be instructed to give a simple hand signal 30 s before each transition whether they can proceed or not. If refusing increased inclination, the participant will be encouraged by testing personnel to continue walking to exhaustion and signal when they want to stop.

Ventilatory and pulmonary gas exchange data will be collected every 10 s during the test. Whether the participant has reached their V̇O_2max_ will be determined by evaluating an eventual plateau of the V̇O_2_ work rate curve in addition to having an RER≥1.05 and/or blood lactate ≥7 mmol/L. V̇O_2max_ will then be calculated as the maximal of the three highest consecutive 10 s values.

#### Secondary outcomes

Maximal lower-leg dynamic muscle strength will be assessed using an inclined leg-press device (h/p/cosmos, Nussdorf-Traunstein, Germany) and defined as the highest load (kg) the participant can handle for one repetition maximum (1 RM) using a standardised protocol.^41^ Testing procedure involves (a) warm-up by performing 5–10 repetitions at 40%–60% of assumed 1 RM, (b) preparation by performing 3–5 repetitions at 60%–80% of assumed 1 RM, (c) testing of maximal strength by consecutively increasing the load (3–5 times) and having the participant attempting 1 repetition with 3 min of rest between each attempt, until failure. Participants are here instructed to position the legs in a 90° knee angle and to mobilise maximal force during the concentric movement phase. The highest achieved load is recorded. This exact testing procedure is repeated to also assess maximal upper-body dynamic muscle strength during a bench-press exercise assisted by a Pivot H3130 Smith machine (Pivot Fitness, Houten, Netherlands). Following the maximal strength testing, participants will rest for 3–4 min and proceed with muscular power (force×velocity) testing using a MuscleLab linear encoder (Ergotest Innovation AS, Porsgrunn, Norway) attached to the leg-press and bench-press devices. The resistance is set at 75% of their 1 RM value attained during baseline testing, and this exact load value is then used in muscle power testing throughout all consecutive follow-up testing phases.

Waist circumference will be assessed using a standardised protocol; measurement is performed at the umbilical level (between crista iliaca and the lower rib) using a measurement tape. Measurement is recorded when the measurement tape is correctly placed and the participant has breathed out. Body weight is measured without outdoor clothing or shoes, using a SECA 770 personal weight (Seca GmbH & CO. KG, Hamburg, Germany).

Device-measured physical activity will be collected using hip-worn triaxial accelerometers ActiGraph wGTX-BT accelerometers (Actigraph, Pensacola, Florida, USA) and the wrist-worn Garmin Forerunner 55 activity tracker (listed under intervention element E). During each testing phase, participants will be instructed to wear the ActiGraph on their right hip for seven consecutive days while awake and only to remove it during activities requiring water contact, such as showering and swimming. Actigraph data will be analysed with the ActiLife software (Actigraph) using a data-driven approach to investigate the influence of different cut-points, epoch lengths and raw accelerometer data, given the lack of international consensus and standard approaches on how to analyse accelerometer data. The Garmin 55 activity tracker is continuously worn from baseline and throughout the study. These data are extracted from the mSpider data collection tool and exported to .csv files for analysis.

#### Tertiary outcomes

Body composition will be assessed with dual-energy X-ray absorptiometry technology using a Lunar Prodigy Advance device (GE Medical Systems, Madison, Wisconsin, USA). Measurements will be performed according to a standardised protocol established by GE Medical Systems. The participant is placed on the device in a supine position with arms placed along the sides. The ‘mirror-mode’ protocol will be applied if the participant’s body size exceeds the scanning area. Total measurement time for a whole-body scan is 8–10 min.

Health-related quality of life will be assessed using the established 5-item EQ-5D-5L instrument, where respondents rate their health in five distinct dimensions including mobility, self-care, usual activities, pain/discomfort and anxiety/depression.^42^

Cognitive function will be measured by the Digit Symbol Substitution Test (DSST), one of the more commonly used neuropsychological tests that requires participants to match symbols to numbers according to a translation key. The DSST is sensitive to cognitive decline by assessing multiple cognitive domains simultaneously.^43^

#### Other outcomes

The RESTART trial will include several additional outcomes. Resting HR and blood pressure will be measured three times in 1-minute intervals after an initial 2-minute rest, using a Welch Allyn Connex ProBP3400 digital (Welch Allyn, Chicago, Illinois, USA) on the right arm, and we will use the mean of the last two blood pressure and HR measurements. We will additionally examine lung function using the Micro 1 Spirometer (CareFusion, Franklin Lakes, New Jersey, USA), with participants exhaling maximally for 6 s during three attempts with 30 s of rest in between.

Muscle strength and physical function will be assessed using clinical measures with established protocols; handgrip strength from a hand-held isokinetic dynamometer and the 5-CST from the Short Physical Performance Battery.^44 45^

We will collect non-fasting venous blood samples using standard methods, including test of thyroid function (thyroxin, thyroid-stimulating hormone), haemoglobin, glycated haemoglobin, blood lipids (total cholesterol, low-density lipoprotein, high-density lipoprotein, triglycerides), liver enzymes (alanine aminotransferase, aspartate aminotransferase, alkaline phosphatase), renal function markers (cystatin C, creatinine), phosphatidylethanol to monitor alcohol consumption, testosterone and C reactive protein. We will additionally ask participants to bring a morning urine sample during testing phases for analysis of the albumin-creatinine ratio.

We will use standardised questionnaires from Tromsø7 to collect data on socioeconomic status (education, household income, occupation status), current social network, ideal weight, chronic diseases, symptoms and complaints, medication use, tobacco use and alcohol intake.^27^ We additionally collect lifestyle and mental health data using the following established questionnaires: Saltin-Grimby Physical Activity Level Scale,^29^ 10-item Hopkins Symptom Checklist,^46^ NORKOST Food Frequency Questionnaire,^47^ 5-item Satisfaction-With-Life Scale,^48^ 10-item General Self-efficacy Scale,^49^ 6-item Self-Compassion Scale,^50^ 10-item Physical Activity Acceptance Questionnaire^51^ and two initial questions from the 4-item Physical Activity Enjoyment Scale.^52^ Emotional and intuitive eating behaviours are assessed by combining the following questionnaires: 3-item Emotional Eating Subscale from the Three-Factor Eating Questionnaire,^53^ one item on binge eating episodes from the Eating Disorder Examination Questionnaire^54^ and 6-item Reliance on Internal Hunger and Satiety Cues Subscale from the Intuitive Eating Scale.^55^

We will explore participants’ experiences in semi-structured interviews to provide a more comprehensive understanding of their experiences and motivation during the trial, and to find potentially important determinants of lifestyle change. These qualitative data can also strengthen our knowledge of potential effects of the trial, as they complement the quantitative information received during the assessment phases and inform of subjective positive health effects attained either in conjunction with, or in the absence of, effects on other physical parameters. Qualitative data will be structured with the QRS NVivo software, and analysed with content- or thematic analyses.^56 57^

Data on resource use will be collected during the trial to facilitate health economic evaluation of the personalised lifestyle programme. In addition, these analyses benefit from the aforementioned collection of health-related quality of life with EQ-5D-5L as recommended for Norwegian health economic evaluations. We will create a Markov model to simulate future participant health based on reported outcomes from the trial to assess the potential long-term health impact of lifestyle changes from the intervention as recommended in international guidelines.^58^ Based on the model, we will also evaluate to what extent the intervention costs will be offset by future cost reductions through reduced health service use. The Markov model will combine future health and resource implications in a health economic evaluation assessing the intervention’s cost-utility compared with the regular HLC lifestyle programme.

Finally, all participants will be followed up for CVD events (myocardial infarction, stroke), diabetes and mortality at 5 years from baseline, by linkage to the Norwegian Patient Registry and the Norwegian Cause of Death Registry.

### Data management

The CTU will use the REDCap tool for collection and management of data at their testing facilities. REDCap is a secure web-based application that supports validated data entry, tracking of all data handling, automated export and manual data import. Data collected using REDCap are intermediately stored on secure research data servers at UiT before being transferred to The Services for Sensitive Data (TSD) at trial close-out. Data collected at the UiT Faculty of Health Sciences research laboratory for sports, physical activity and public health are manually exported from data collection software in comma-separated (.csv) file formats directly into TSD. De-identified physical activity data, continuously collected using the mSpider software, will be stored intermediately in a Microsoft Azure cloud storage solution, managed by RESTART project members, before being transferred to TSD for persistent storage.

### Statistical methods

We will conduct an intention-to-treat (ITT) analysis to evaluate the effects on all outcomes. Our primary analysis will use linear mixed models, which are well-suited for handling missing data under the assumption that missing data are missing at random. This statistical approach allows us to include all available information from participants even if they drop out of the trial, thereby preserving the benefits of randomisation and maintaining the integrity of the ITT principle. The models will include fixed effects for treatment groups and time, as well as random effects to account for within-subject correlation over time. As a sensitivity analysis, we will conduct a per-protocol analysis by excluding participants who drop out of the trial. This analysis will help assess the robustness of our findings under different assumptions about the missing data. We will additionally explore patterns of missing data and perform exploratory analyses to identify potential predictors of missingness. This will help us understand the nature of the missing data and refine our assumptions if necessary.

## Methods: monitoring

### Data monitoring

Trial monitoring will be purchased from the CTU monitor network.

### Harms

Potential adverse events are continuously monitored throughout the study period by the intervention instructors, and systematically registered by assessors during the baseline, 6-, 12- and 24-month testing phases. All potential adverse events occurring during the intervention activities are reported continuously to the study coordinator and the study-associated medical doctor (MD). The MD performs an initial assessment of the adverse event and can during the initial 18 months of the trial consult an MD and/or a physiotherapist with specialisations in sports medicine for eventual second opinions. Potential adverse events will be graded in line with the Common Terminology Criteria for Adverse Events. Participants are informed in the informed consent documentation (see online supplemental file 1) that physical training may lead to anticipated harms such as delayed-onset muscle soreness, pain or repetitive strain injuries. Experiences from the pilot study showed that short-term alternative exercises or adjustments (grip, angle of motion) to current exercises work well in cases of physical training-induced pain and discomfort, and this will be implemented in the RESTART trial.

### Auditing

Potential audits are ordered and executed by the RCN.

## Ethics and dissemination

### Research ethics approval

The RESTART trial was approved by the Regional Committee for Medical Research Ethics in Northern Norway (Reference 584841/2023) in May 2023.

### Protocol amendments

Potential amendments to the study protocol, such as changes to eligibility criteria, outcomes or analyses will need to be approved by the RESTART steering committee and discussed with the scientific advisory board. Major amendments are communicated to the regional ethics committee, trial sponsors, outcome assessors and trial participants.

### Consent or assent

Written informed consent (or assent) is provided by the participants and collected by a research nurse at the CTU when participants arrive for baseline assessment. Signed informed consent documentation is collected by the study coordinator for scanning and storage in TSD. A translated example (original language: Norwegian) of the informed consent form is available as online online supplemental file 01.

### Confidentiality

Potential participants will be identified in the Tromsø Study by extracting data on inclusion criteria variables and contact information (name, telephone, living address). Enrolled participants are given a unique trial ID to be used by all personnel throughout the study. Personnel delivering the intervention elements are allowed to know the participant’s surname. The linkage between each participant’s personal identification number, contact information and the trial ID will be securely stored in an ISO 27001-certified online data platform (Helseboka) developed by Norwegian health authorities during the COVID-19 pandemic. The UiT uses a Microsoft SharePoint solution where local RESTART databases containing study documents are protected by two-factor authentication and Microsoft Azure Information Protection protocols, restricting access only to study management members. Participant contact information will be shared with the research nurse at the CTU responsible for coordinating baseline assessments. The unidentified dataset will not be made publicly available to prevent reverse identification of participants.

### Access to data

The principal investigators will have access to the final, complete trial dataset. Other investigators can apply to the steering committee for data and may receive access to data with variables specific to the research question of interest.

### Ancillary and post-trial care

Any participants who suffer injuries from trial participation are eligible for compensation from The Norwegian System of Patient Injury Compensation. UiT is additionally self-insured and retains the risk of loss itself rather than transferring that risk to an insurance company.

### Dissemination policy

The target audiences and stakeholders/users of the project are the ageing population (study participants), their families, and the municipalities and counties responsible for public health surveillance and initiatives, NGOs, health and fitness centres, the general public and the scientific community.

The different communication activities during the trial timeline will be coordinated on the communication platform being developed in the ongoing RCN-funded project ‘Healthy choices and the social gradient’ (RCN ES617148/289440). Target groups and communication aims will differ throughout the project timeline (table 1). Early phase communication activities aim to recruit study participants and support participant interaction and compliance. Later phases will focus on communicating results to the ageing population, the scientific community, municipal primary healthcare, health authorities and the general public. Exploitation measures include (a) in 2024: develop and communicate self-selected physical training options with project collaborators; (b) from 2025: establish RESTART workshops involving participants and project partners to develop and communicate project outputs to target audiences and stakeholders; (c) in 2026: establish working groups of researchers, municipal primary healthcare, health authorities, NGOs and participants to develop a manual for a stepwise implementation of the RESTART in Norwegian municipalities (separate funding to be sought).

**Table 1:** Dissemination activities

Year	Target audience	Aims	Measures
2023–2026	Study participants	Recruit study participants, support participant interaction and compliance, communicate self-selected physical training options	Media coverage, Tromsø Study webpages, RESTART eTool, healthy choices communication platform, social media for study participant interaction
2025–2027	The ageing population	Communicate research findings and tools	RESTART eTool, healthy choices communication platform, municipality and NGO’s communication channels to distribute info, blog, podcasts, short videos, infographics
2025–2027	The scientific community	Discuss and share research findings	Conference presentations, peer-reviewed publications (prioritise Open Access)
2026–2027	Municipalities, counties, health authorities and the general public	Inform of project output, engage in dialogue, make tools available	Working groups to develop manual for implementation, upscaling of RESTART eTool, self-selected physical training options, communicating via channels: municipality, NGOs, the helsenorge.no platform

NGO, non-governmental organisation; RESTART, RE-inventing Strategies for healthy Ageing: Recommendations and Tools.

For scientific dissemination, authorship eligibility will be based on guidelines established in the International Committee of Medical Journal Editors (ICMJE). No use of professional writers is intended. Due to privacy concerns, there are no plans to provide the dataset to the public, although the full protocol and statistical analysis code will be available on request.

## Discussion

The main objective of the RESTART trial is to investigate whether we can achieve lasting lifestyle change in a population of older adults with cardiometabolic risk. To achieve this aim, we have designed a complex prevention model with long-term follow-up, focusing on key lifestyle behaviours (supervised high-intensity training, diet counselling, behaviour counselling) and gradually supporting the participants to become self-sufficient in maintaining their improved health. The success of the trial is likely dependent on several important factors occurring during the different trial phases. Some may be exemplified as (a) an improved sense of mastery and well-being in everyday life among participants, through the anticipated physical form improvements, reduced adiposity and identification of person-centered values and goals, (b) establishment of new social contexts and sense of social commitment through the group-based intervention sessions, (c) the efficacy of the prevention model design, which gradually enables participant independence in maintaining a healthier lifestyle.

However, the RESTART trial may also face several challenges that may affect project outcomes and their generalisability to the broader population. Despite our use of inclusion criteria to recruit older adults with cardiometabolic disease risk, there is always the possibility of selection bias occurring within this population segment due to socioeconomic and cultural factors, health status, and motivation for lifestyle change. Complete blinding in exercise interventions is difficult, because while we can conceal the group allocation for outcome assessors, it is impossible to blind the participants as they will know whether they receive the complex lifestyle intervention or not. Because of the relatively long duration of the trial (24 months), we can also expect a degree of variability in participant adherence due to gradual changes in motivation and health factors that complicate further participation. Another challenge is the scalability of the trial and the potential implementation of study results into municipalities and NGOs. Conducting a long-term complex intervention programme requires significant costs and resources, and increasing scalability might require decreased measurement precision when evaluating outcomes, and fewer intervention activities. This may have consequences for the implementation and reproducibility of the RESTART trial’s main results in communities and municipalities.

## Supplementary material

10.1136/bmjopen-2024-095810 online supplemental file 1

10.1136/bmjopen-2024-095810 online supplemental file 2
