# Supplementary material for: Improving and preserving cardiorespiratory fitness, muscle strength and adiposity through a complex lifestyle intervention in community-dwelling older adults with elevated cardiometabolic risk: study protocol for the RESTART randomised controlled trial
Source: BMJ Open. 2025 Apr 19;15(4):e095810. doi: 10.1136/bmjopen-2024-095810 (PMC12010311; doi:10.1136/bmjopen-2024-095810)
Supplement: online supplemental file 1 [file bmjopen-15-4-s001.docx]

**Supplementary information 01**

DO YOU WANT TO PARTICIPATE IN THE RESEARCH PROJECT restart?

## The purpose of the project and why you are asked

This is a question for you to participate in a research project that will investigate the long-term effect of a complex intervention, RESTART, to prevent disease among older adults with an increased risk of disease. RESTART intends to test the effect of physical activity and dietary interventions among people who have demonstrated an elevated risk of cardiovascular disease, either in the seventh Tromsø Study (Tromsø7), or with their GP.

Sedentary lifestyle, overweight and obesity increase the risk of disease. Several studies show that physical activity reduces the risk of cardiovascular disease and diabetes. However, few studies have shown long-term effects of physical activity, dietary interventions and motivational interviewing in people with an increased risk of cardiovascular disease. We have previously gathered experience and investigated the feasibility of RESTART in a feasibility study that included 16 people. We will now investigate whether older adults can reduce the risk of disease through increased physical activity, improved fitness, muscle strength, and dietary habits. We will also investigate the effect on quality of life. Study participants are randomly assigned to an intervention or control group and followed up over a period of two years.

RESTART is carried out by UiT The Arctic University of Norway in collaboration with Tromsø municipality, the University Hospital of North Norway (UNN), the National Association for Heart and Lung Disease (LHL), Troms Turlag, the Centre for Morbid Obesity in the South-Eastern Norway Regional Health Authority and Akershus University Hospital. UiT The Arctic University of Norway is responsible for the personal data processed in the project.

## What does the PROJECT mean for you?

To participate in RESTART, you must have participated in the seventh Tromsø Study and have been shown to have an elevated risk of cardiovascular disease, obesity and low levels of physical activity. You can also get an invitation through your GP. You must be between 60-75 years old, have a body mass index (BMI) of 28 kg/m2 or more and have an increased risk of cardiovascular disease. You must have a smartphone and bankID and also be motivated and consent to participate. You cannot have a serious illness or illness that limits your participation in project activities.

After a telephone interview, people who wish to participate in RESTART will be invited to meet at the Clinical Trial Unit at UNN to investigate whether they meet the requirements to participate in the research project. In this case, you will be asked to sign your consent to participate. You will then fill out a questionnaire and be examined at the Clinical Trial Unit. You will also undergo strength and conditioning tests to examine physical fitness. You are then randomly assigned to an intervention or a control group. Both groups receive updated advice on health-promoting lifestyles and an activity tracker for continuous measurement of physical activity. Both groups will be called in for examinations at the Clinical Trial Unit at UNN, and for strength and fitness tests after 6, 12 and 24 months until 2026. If the project receives funding, participants are invited to a follow-up survey with a questionnaire in 2027.

**Schedule of the study**

**Intervention group only**

This group participates in a training program at KRAFT sports center. The activity includes guided strength and cardio training in groups twice a week from ....... o'clock until....... over a period of one and a half years. The training will take place both outdoors and inside the hall at KRAFT. The activities are led by experienced instructors, and will eventually be taken over by the Wellness Center in Tromsø municipality. The instructors will use participants' first names unless they opt out of this. Participants are encouraged and guided to self-activity in addition to the organized activity. After a year and a half, study participants are encouraged to engage in physical activity of their own choosing.

After 3-6 months in the study, time will be set aside for dietary guidance and motivational group discussions at KRAFT. The intervention group is also offered a tailor-made online tool to strengthen health-promoting habits.

*Interview*

After 12 and 24 months, study participants in the intervention group will be asked for verbal interviews. The conversations are recorded on tape, printed out and then analyzed. The tape is deleted when the interviews are printed. All information is treated confidentially, and all presentation of results will take place in anonymized form. When all interviews have been analyzed, all interview data will be deleted. The interviews will last approx. 1 hour each time.

**Both intervention and control group**

*Surveys and questionnaires*

All study participants are summoned to the Clinical Trial Unit at UNN at the start of the study and after 6, 12 and 24 months in the study. Height/weight, hip/waist circumference, blood pressure, heart rate, ECG, lung function and body composition are measured. Simple tests will also be carried out on everyday functioning, a cognitive test and blood and urine samples will be taken. If you have abnormal blood tests or results from examinations, the study management will contact you and possibly your GP to inform you about this. All participants complete a questionnaire at the start of the study and after 12 and 24 months in the study. The visits to the Clinical Trial Unit and the completion of the questionnaire will take a total of 2-3 hours.

*Blood and urine tests*

Blood samples are taken from all study participants to examine blood count, blood lipids (cholesterol and triglycerides), long-term blood glucose (HbA1c) and kidney function at study entry and after 6, 12 and 24 months. Blood tests to examine liver function, marker of inflammation (CRP), alcohol intake (PEth), and testosterone are taken at study entry and after 12 and 24 months. Metabolism tests are only taken at the start of the study. Urine samples are also taken to examine kidney function.

*Fitness test*

All study participants complete a test to measure maximal oxygen uptake (VO2max) during physical exertion at the start of the study, and after 6, 12, and 24 months in the study. The test begins with a 20-minute gentle warm-up on a treadmill or on an ergometer. The test itself takes about 8 minutes and takes place by the participant walking/running on a treadmill. During the test, heart rate, oxygen uptake (O2) and ventilation of carbon dioxide (CO2) are measured by the participant breathing through a tube with a mouthpiece. The test is not dangerous, but can be experienced as a little uncomfortable if the participant is not used to pushing themselves. Test participants are encouraged to perform to the maximum, but can end the test at any time if they are unable to do more. The fitness and strength test takes approx. 1 hour each time and takes place at UiT.

*Activity tracking*

All study participants are given an activity tracker (Garmin®, Forerunner55), a watch that should be worn most of the day throughout the study period. This is preset with a fictitious identity that is linked to the participants' number in RESTART to safeguard the participants' privacy and ensure that we collect data that can be used to calculate sleep, sedentary lifestyle, heart rate and physical activity to the study's own data storage server at UiT. If participants change the settings on the watch, it may prevent us from collecting the data we want, and we therefore request that participants do not use the activity tracker in any other way than agreed with the project manager or project staff. GPS must be turned off for the entire study period.

The watch must be connected to the participant's personal smartphone (Android/iPhone). Privacy cannot be guaranteed vis-à-vis Garmin (the watch) or Apple/Google (smartphone). Garmin accesses activity data recorded by your watch. UiT is not responsible for the processing of personal data by Garmin, Google, or Apple. For more information about Garmin's privacy policy, see <https://www.garmin.com/nb-NO/privacy/global/>.

At study entry and after 12 and 24 months in the study, participants are asked to wear activity trackers (Actigraph) that are worn on the hip in a belt around the clock for one week and then returned to the study management.

## Possible advantages and disadvantages

Physical activity is believed to have many positive effects on health. Physical activity has been shown to prevent cardiovascular disease. Being physically active increases well-being, well-being and mental health, and thus increases quality of life.

Participants may experience soreness and pain in the muscles after exercise. This is harmless, but can be a little uncomfortable. At the start of exercise, some people may experience joint pain as a result of unaccustomed strain. It is then important to provide information about this so that the amount of strain can be adjusted.

When people who have been not physically active increase their activity, there is a certain risk of getting repetitive strain injuries. To avoid this, we in the intervention group will start from the individual participant's endurance, strength and mobility. If the symptoms persist, we will refer for assessment to a project-affiliated doctor and possibly a physiotherapist. Expenses for the consultation are charged to the project and information about the consultation is forwarded to the participant's GP.

## Voluntary participation and the possibility of withdrawing consent

Participation in the project is voluntary. If you wish to participate, please sign the declaration of consent on the last page. You may withdraw your consent at any time and without giving any reason. There will be no negative consequences for you if you do not want to participate, or later choose to withdraw. If you withdraw your consent, no further research will be conducted on your information and your biological material. You can demand access to the information stored about you, and this will then be disclosed within 30 days. You can also demand that your data in the project be deleted and that the biological material be destroyed.
The right to demand destruction, deletion or disclosure does not apply if the material or information has been anonymized or published. This access may also be restricted if the information has been included in analyses carried out.

If you later wish to withdraw or have questions about the project, you can contact the project manager or study coordinator (see contact information on the last page).

## What happens to the INFORMATION about you?

The information registered about you will only be used as described under the purpose of the project, and is planned to be used until 31.12.2030. We process the personal data confidentially and in accordance with the data protection regulations. The information about you will be compared with information you have previously provided about risk factors for cardiovascular disease in the Tromsø Study. We will also compare the information with data from the Norwegian Patient Registry and the Cause of Death Registry. Any extensions in use and storage period can only take place after approval from the Regional Ethics Committee and other relevant authorities. At the end of the project, your data will be returned to the Tromsø Study Health Registry. You have the right to access what information is registered about you and the right to have any errors in the information that is registered corrected. You also have the right to access the security measures when processing the data. You can complain about the processing of your data to the Norwegian Data Protection Authority and UiT's data protection officer. You can find contact information for the data protection officer further down in the information letter.

Everyone involved in the project is subject to a duty of confidentiality. All information and samples will be processed without name and national identity number or other directly recognizable information. A code links you to your information and samples through a list of names, and this list of codes will be stored electronically and inaccessible to unauthorized persons. Only project manager Sameline Grimsgaard and researcher Jonas Johansson have access to this list. It will not be possible to identify you in the results of the study when these are published.

All publication must be done in such a way that individual participants cannot be recognized, but we are obliged to inform you that we cannot rule out that this may happen.

After the research project has been completed, the information about you will be stored for five years for control purposes.

## What happens to samples that are taken from you?

The blood samples that are taken are continuously analyzed with regard to blood percentage, kidney and liver function, muscle enzymes, long-term blood sugar (HbA1c) and the level of fats in the blood. Blood samples for analysis of inflammatory and biomarkers and urine for analysis of kidney function will be frozen and stored in a research biobank associated with the project. The biobank is located at the Faculty of Health Sciences at UiT The Arctic University of Norway. The samples are analyzed together before the end of the project. The biobank will cease to exist at the end of the project on 31.12.2030 and the remaining blood and urine will be destroyed.

## Insurance

The university is a self-insurer and will handle damages and losses that may occur in the project.

## Follow-up PROJECT

If the project receives funding, participants may be invited to a follow-up survey in 2027.

## Economy

The study is funded by the Research Council of Norway (KSP 336341), the Faculty of Health Sciences, UiT The Arctic University of Norway and the Simon Fougner Hartmann Family Fund. The National Association for Heart and Lung Disease, Troms Hiking Association and the Centre for Morbid Obesity in the South-Eastern Norway Regional Health Authority contribute with their own efforts. The online tool for health-promoting habits has been developed with support from the pharmaceutical company Novo Nordisk. There are no conflicts of interest in the project.

Participation in the study is free of charge. The project covers the costs of training at KRAFT during the intervention period of 18 months. Participants must cover the expenses for transport to KRAFT. The use of public transport in connection with visits to the Clinical Trial Unit and for fitness tests at UiT is covered by the study in the form of a gift card of NOK 600, which is handed out at the start of the study. Participants will be given an activity tracker for use in the project.

## Approvals

The project has been approved by the Regional Committee for Medical and Health Research Ethics (REK NORD) no. 584841.

## CONTACT DETAILS

If you have any questions about the project, experience adverse events or side effects, or wish to withdraw from participation, please contact:

The RESTART project, tel. 920 80 494, e-mail [restart@helsefak.uit.no](mailto:restart@helsefak.uit.no)

Project manager Professor Sameline Grimsgaard, tel. 913 51 858, e-mail [sameline.grimsgaard@uit.no](mailto:sameline.grimsgaard@uit.no)

Researcher Jonas Johansson, tel. 934 62 934, e-mail [jonas.johansson@uit.no](mailto:jonas.johansson@uit.no) , or

Project coordinator Sanne Melum, tel. 920 80 494, e-mail [sanne.k.melum@uit.no](mailto:sanne.k.melum@uit.no)

UiT The Arctic University of Norway's Data Protection Officer can be reached by e-mail [personvernombud@uit.no](mailto:personvernombud@uit.no)or phone: 77 64 69 52.

# Consent to participate in the PROJECT "RESTART" 2024-2027

## I am willing to participate in the project

| Place and date | Participant's signature |
| --- | --- |
|  |  |
|  | Participant's name in printed letters |

I confirm that I have provided information about the project

| Place and date | Signature |
| --- | --- |
|  |  |
|  | Role in the project |
