# Supplementary material for: Improving and preserving cardiorespiratory fitness, muscle strength and adiposity through a complex lifestyle intervention in community-dwelling older adults with elevated cardiometabolic risk: study protocol for the RESTART randomised controlled trial
Source: BMJ Open. 2025 Apr 19;15(4):e095810. doi: 10.1136/bmjopen-2024-095810 (PMC12010311; doi:10.1136/bmjopen-2024-095810)
Supplement: online supplemental file 2 [file bmjopen-15-4-s002.docx]

**Supplementary information 02**

1. Thyroid dysfunction (thyroxine <7 pmol/L and thyroid-stimulating hormone >10 mIU/L, and/or thyroxine >25 pmol/L and thyroid-stimulating hormone <0.2 mIU/L)
2. Liver dysfunction (alanine aminotransferase >210 µmol/L for men, >135 µmol/L for women, and/or aspartate aminotransferase >135 µmol/L for men, >105 µmol/L for women, and/or alkaline phosphatase >345 µmol/L)
3. Kidney dysfunction (creatinine >220 µmol/L), in addition to prognosis of CKD by GFR and Albuminuria categories (KDIGO 2012, Levey et al. 2020)
4. Severe anemia (hemoglobin <10g/dL for men, <8g/dL for women)
5. Uncontrolled diabetes (glycated hemoglobin ≥86 mmol/mol)
