# Supplementary material for: Improving and preserving cardiorespiratory fitness, muscle strength and adiposity through a complex lifestyle intervention in community-dwelling older adults with elevated cardiometabolic risk: study protocol for the RESTART randomised controlled trial
Source: BMJ Open. 2025 Apr 19;15(4):e095810. doi: 10.1136/bmjopen-2024-095810 (PMC12010311; doi:10.1136/bmjopen-2024-095810)
Supplement: Supplementary file 3 [file bmjopen-2024-095810.reviewer_comments.pdf]

PEER REVIEW HISTORY

BMJ Open publishes all reviews undertaken for accepted manuscripts. Reviewers are asked to complete a checklist review form and are provided with free text boxes to elaborate on their assessment. These free text comments are reproduced below.

ARTICLE DETAILS

Title (Provisional)

Improving and preserving cardiorespiratory fitness, muscle strength and adiposity through a complex lifestyle intervention in community-dwelling older adults with elevated cardiometabolic risk: study protocol for the RESTART randomized controlled trial

Authors

Johansson, Jonas; Deraas, Trygve Sigvart; Hopstock, Laila Arnesdatter Arnesdatter; Henriksen, André; Grimsgaard, Sameline

---

VERSION 1 - REVIEW

**Reviewer**	**1**
**Name**	**REDDY, Ravi**
**Affiliation**	**King Khalid University, Department of Medical Rehabilitation Sciences, King Khalid University, Abha, Saudi Arabia**
**Date**	**18-Jan-2025**
**COI**	**None**

Reviewer’s Report

Abstract

The abstract could benefit from greater precision and clarity in presenting the study’s design and methodology. While the primary outcome is mentioned, secondary and tertiary outcomes, as well as their relevance, are insufficiently detailed. Additionally, the abstract lacks explicit mention of the study’s limitations, such as the inability to detect changes in hard endpoints (e.g., myocardial infarction or mortality), which could contextualize the research scope. The description of the interventions is broad; a concise summary of key intervention components (e.g., exercise regimen, behavioral therapy, dietary guidelines) would provide more clarity.

Introduction

The introduction could improve by offering a more critical analysis of gaps in the existing literature on lifestyle interventions for older adults. While the manuscript highlights the

aging population and associated cardiometabolic risks, it does not delve into why previous interventions have failed to achieve long-term behavior change or how the RESTART study specifically addresses these gaps. The rationale for focusing on a specific age group (60-75 years) and exclusion criteria (e.g., dementia, mobility issues) is insufficiently justified. Additionally, there is limited discussion on potential barriers to implementation and adherence, such as cultural or socioeconomic factors, which may influence the generalizability of findings.

Methods

The methods section lacks sufficient detail on the randomization process, specifically how allocation concealment and stratification by sex will be operationalized. The inclusion and exclusion criteria, though comprehensive, are not adequately justified; the rationale for specific thresholds (e.g., BMI ≥ 30 kg/m² or NORRISK 2 score) is not discussed. Greater transparency is needed regarding the intervention's scalability and the potential impact of the complex, multi-sectoral design on real-world implementation. Moreover, the statistical analysis plan does not outline how missing data will be handled beyond intention-to-treat analyses. The authors could also elaborate on the rationale behind specific secondary and tertiary outcome measures, such as cognitive function and quality of life, to connect them to the primary objective.

Results

Although this is a protocol, the anticipated results section could be more robust. There is minimal discussion on potential confounding variables and how these will be controlled or adjusted in the analyses. The authors should address the expected variability in adherence to the intervention and its implications for study outcomes. Additionally, the methods for monitoring intervention fidelity, particularly for elements transitioned to external providers (e.g., municipal health centers), remain unclear. Greater emphasis on how qualitative data will complement quantitative findings and inform determinants of lifestyle change would strengthen the study's potential impact.

Discussion

The discussion section does not adequately consider the potential challenges of conducting a long-term, complex intervention in this population, such as attrition, motivational decline, or external factors (e.g., unforeseen health events in participants). There is limited exploration of how the study results could inform public health policy or contribute to guidelines for cardiometabolic risk prevention. The authors should also discuss how the findings will be interpreted in the context of existing evidence and what unique contributions the study will make. Furthermore, potential biases, such as self-selection bias and the lack of blinding for participants, are not sufficiently acknowledged.

Conclusion

The conclusion does not provide actionable insights into how the study findings will be utilized or disseminated beyond academic channels. A stronger emphasis on the potential scalability of the intervention and how it aligns with broader public health initiatives would improve this section. Additionally, the conclusion fails to critically address the limitations of the study design, such as the small sample size relative to the expected attrition or the exclusion of participants without access to technology (e.g., smartphones for the eTool component), which could limit generalizability.

**Reviewer**	**2**
**Name**	**Weston , Kathryn**
**Affiliation**	**University of Strathclyde**
**Date**	**07-Feb-2025**
**COI**	**None**

Thanks you for the opportunity to review this study protocol. The protocol is well written, very thorough and clearly describes the processes in place for the RESTART trial. I have some minor comments and suggestions for the authors to consider, which are detailed below.

Page 9, Line 35. Re. physical activity devices given prior to randomisation. What will happen if the tracker data indicates the participant is indeed active? Will they be excluded from the trial for example? If so what cut-points etc will be used to determine PA levels here?

Page 10, Line 10-35. The authors should be commended on providing detailed information on what their exercise protocol will consist of. Could they also provide detail on how the exercise will be progressed over time? I also suggest adhering to the consensus of exercise reporting template when describing their exercise component, to ensure replication by others if the study is successful. The authors may also want to consider how they will assess the fidelity of their exercise intervention. References to support both suggestions are detailed below,

Slade SC, Dionne CE, Underwood M, Buchbinder R. Consensus on exercise reporting template (CERT): explanation and elaboration statement. British journal of sports medicine. 2016 Dec 1;50(23):1428-37.

Taylor KL, Weston M, Batterham AM. Evaluating intervention fidelity: an example from a high-intensity interval training study. PloS one. 2015 Apr 22;10(4):e0125166.

Line 47. Could some more detail be provided on what the functional exercises will consist of?

Line 59. Re. “These organizations are encouraged to align their activities with the training principles of the RESTART trial.” – can some detail be provided on how this encouragement will be done and monitored over time? I.e. will the organisations receive training from the RESTART trial group?

Page 11. Line 49. Can more detail be provided on the etool? Is this a smartphone application for example? And how has this been developed?

Page 12. Lines 34 onwards. The authors should be commended for detailing how they intend to avoid, monitor and report adverse events. In line with recent guidance, I suggest they consult and adhere to the CONSORT Harms guidance.

Junqueira DR, Zorzela L, Golder S, Loke Y, Gagnier JJ, Julious SA, et al. CONSORT Harms 2022 statement, explanation, and elaboration: updated guideline for the reporting of harms in 1090 randomised trials. BMJ. 2023 Apr 24;381:e073725

Page 12. Line 55-56. Could the authors please expand on how they intend to assess/ monitor the fidelity of the intervention? Which markers will be used to assess fidelity for example (i.e., heart rate) and will this also include researcher observations/ checklists etc?

Page 16. Line 53. Please consider changing ‘objective physical activity’ to device-measured physical activity, here and throughout the manuscript. This is to acknowledge that a degree of subjectivity still exists when using devices to measure physical activity (e.g., which cut points are applied, epoch length etc), and the measurement is therefore not truly objective. Please also add detail on how the physical activity data will be analysed.

VERSION 1 - AUTHOR RESPONSE

Reviewer: 1
Dr. Ravi REDDY, King Khalid University
Comments to the Author:
Reviewer’s Report

Abstract

The abstract could benefit from greater precision and clarity in presenting the study's design and methodology. While the primary outcome is mentioned, secondary and tertiary outcomes, as well as their relevance, are insufficiently detailed. Additionally, the abstract lacks explicit mention of the study's limitations, such as the inability to detect changes in hard endpoints (e.g., myocardial infarction or mortality), which could contextualize the research scope. The description of the interventions is broad; a concise summary of key intervention components (e.g., exercise regimen, behavioral therapy, dietary guidelines) would provide more clarity.

Response: Thank you for taking the time to review our manuscript.

We agree that we could improve the abstract by being more specific on the intervention and outcome details. As such, we have now adjusted and expanded the abstract to accommodate this and provide more information regarding the intervention characteristics and relevance of the outcomes (page 2). We have not included study limitations specifically in the abstract text, as this is already included in a separate section immediately after the abstract (required by BMJ Open manuscript formatting guidelines). We hope that is satisfactory to the reviewer.

Introduction

The introduction could improve by offering a more critical analysis of gaps in the existing literature on lifestyle interventions for older adults. While the manuscript highlights the aging population and associated cardiometabolic risks, it does not delve into why previous interventions have failed to achieve long-term behavior change or how the RESTART study specifically addresses these gaps. The rationale for focusing on a specific age group (60-75 years) and exclusion criteria (e.g., dementia, mobility issues) is insufficiently justified. Additionally, there is limited discussion on potential barriers to implementation and adherence, such as cultural or socioeconomic factors, which may influence the generalizability of findings.

Response: Thank you for these suggestions to improve the introduction section. We have now revised and expanded the introduction (page 4 and 5), elaborating on existing literature to be more specific on knowledge gaps and potential approaches in relation to complex lifestyle interventions in older adults, and how the RESTART trial can potentially address this. Regarding the rationale for the inclusion/exclusion criteria, we have tried addressing this in relation to your comments on the inclusion/exclusion criteria in the Methods section (page 7-8) further below, where these criteria are first mentioned. We also agree that we could expand the discussion on potential implementation and adherence barriers in our study. We felt it was appropriate to address this in the newly added Discussion section (page 24) in relation to your comments further below, and hope this is satisfactory.

Methods

The methods section lacks sufficient detail on the randomization process, specifically how allocation concealment and stratification by sex will be operationalized. The inclusion and exclusion criteria, though comprehensive, are not adequately justified; the rationale for specific thresholds (e.g., BMI ≥ 30 kg/m^2 or NORRISK 2 score) is not discussed. Greater transparency is needed regarding the intervention's scalability and the potential impact of the complex, multi-sectoral design on real-world implementation. Moreover, the statistical analysis plan does not outline how missing data will be handled beyond intention-to-treat analyses. The authors could also elaborate on the rationale behind specific secondary and tertiary outcome measures, such as cognitive function and quality of life, to connect them to the primary objective.

Response: We have now provided more details on the allocation, concealment mechanism, and randomization process, under the "Allocation" section, on page 14-15. We have additionally expanded the "Eligibility criteria" section (page 7) to provide more rationale for the used inclusion/exclusion criteria, to accommodate the reviewer's comments raised here and in the previous comment on the introduction. We are a bit unsure whether a more transparent discussion on scalability and potential impact would fit within the Methods section of the manuscript and have opted instead to include this in the newly added Discussion section (page 24) – we hope this is satisfactory. We agree that the statistical analysis plan required more information on how we plan to handle missing data, and we have now expanded the "Statistical methods" section accordingly (page 20). Lastly, we have also expanded upon the "Outcomes" section (page 12-13) in relation to the reviewer's comment on clarifying the rationale behind the secondary and tertiary outcome measures. To summarize, we thank the reviewer for providing us with these comments which we believe have helped improve the manuscript's readability.

Results

Although this is a protocol, the anticipated results section could be more robust. There is minimal discussion

on potential confounding variables and how these will be controlled or adjusted in the analyses. The authors should address the expected variability in adherence to the intervention and its implications for study outcomes. Additionally, the methods for monitoring intervention fidelity, particularly for elements transitioned to external providers (e.g., municipal health centers), remain unclear. Greater emphasis on how qualitative data will complement quantitative findings and inform determinants of lifestyle change would strengthen the study's potential impact.

Response: We are a bit unsure about the initial comment as there was no anticipated results section included in our manuscript. Such a section is not required according to the BMJ Open formatting guidelines for study protocols or in the SPIRIT guidelines, which we followed closely when developing this manuscript. We are also hesitant to add discussion on potential confounding, as this is a study protocol for a randomized controlled trial, where confounding is much less of an issue compared to observational studies due to randomization. In line with the reviewers' previous comments regarding discussion of adherence and implications, we have chosen to add this to the newly added Discussion section (page 24), as we feel that speculations are better suited there than in the Methods section.

We agree that we could have been clearer about monitoring intervention fidelity and have tried expanding this under the newly added "Adherence and fidelity" section on page 12. It should be noted that fidelity is more closely followed-up the first 12 months during the project-delivered supervised intervention sessions. At 12 months the participants are transitioned to self-selected training and is supposed to follow the exercise regimen on their own based on improved health literacy, physical form, and motivation attained during the first 12 months. Thus, to follow the trial protocol and aim, we lessen the follow-up of fidelity at this point.

We have also, per the reviewer's suggestion, tried expanding on how the qualitative assessments strengthen our understanding of the potential lifestyle effects of the trial (page 19).

Discussion
The discussion section does not adequately consider the potential challenges of conducting a long-term, complex intervention in this population, such as attrition, motivational decline, or external factors (e.g., unforeseen health events in participants). There is limited exploration of how the study results could inform public health policy or contribute to guidelines for cardiometabolic risk prevention. The authors should also discuss how the findings will be interpreted in the context of existing evidence and what unique contributions the study will make. Furthermore, potential biases, such as self-selection bias and the lack of blinding for participants, are not sufficiently acknowledged.

Response: Again, we are a bit unsure about the initial comment, as we did not originally include a discussion section because it was not recommended in the SPIRIT and BMJ Open guidelines when formatting a study protocol. However, we believe the reviewer raises important points and we think that such a section can strengthen the manuscript. A new Discussion section (page 24) has thus been added, which considers several of the potential challenges facing the trial, in line with comments raised by the reviewer in this and the previous sections.

Conclusion
The conclusion does not provide actionable insights into how the study findings will be utilized or disseminated beyond academic channels. A stronger emphasis on the potential scalability of the intervention and how it aligns with broader public health initiatives would improve this section. Additionally, the conclusion fails to critically address the limitations of the study design, such as the small sample size relative to the expected attrition or the exclusion of participants without access to technology (e.g., smartphones for the eTool component), which could limit generalizability.

Response: Thank you for the comment. It was, however, pointed out to us by the editor that a conclusion section is not part of the BMJ Open formatting requirements for study protocols and we were asked to not include this in the manuscript. Therefore, no such section has been added.

Reviewer: 2
Dr. Kathryn Weston , University of Strathclyde
Please also see attached file.

Comments to the Author:

Thanks you for the opportunity to review this study protocol. The protocol is well written, very thorough and clearly describes the processes in place for the RESTART trial. I have some minor comments and suggestions for the authors to consider, which are detailed below.

Response: Thank you for the encouraging words and for taking the time to review our manuscript.

Page 9, Line 35. Re. physical activity devices given prior to randomisation. What will happen if the tracker data indicates the participant is indeed active? Will they be excluded from the trial for example? If so what cut-points etc will be used to determine PA levels here?

Response: Thank you for the insightful comment. We have opted to rely on the self-reported physical activity level for inclusion, as this information relates to the participants overall activity habits during the past year while devices only capture a short activity window and may be subjected to temporary fluctuations, as potential participants become motivated to change their lifestyle as they are recruited into the study. There is thus no plan to exclude participants based on tracker data, although we agree this could have been a valuable addition to the screening process.

Page 10, Line 10-35. The authors should be commended on providing detailed information on what their exercise protocol will consist of. Could they also provide detail on how the exercise will be progressed over time? I also suggest adhering to the consensus of exercise reporting template when describing their exercise component, to ensure replication by others if the study is successful. The authors may also want to consider how they will assess the fidelity of their exercise intervention. References to support both suggestions are detailed below,
Slade SC, Dionne CE, Underwood M, Buchbinder R. Consensus on exercise reporting template (CERT): explanation and elaboration statement. British journal of sports medicine. 2016 Dec 1;50(23):1428-37.
Taylor KL, Weston M, Batterham AM. Evaluating intervention fidelity: an example from a high-intensity interval training study. PloS one. 2015 Apr 22;10(4):e0125166.

Response: Thank you for the positive feedback and constructive suggestions. We have now added information on how the machine-based and functional exercise sessions will be progressed (page 9). Additionally, we have tried adjusting and adding information (page 8-9) when describing the training intervention to better align with the different parts raised in the CERT.

We have also, in line with the comment from Reviewer #1, added information on how we will assess fidelity during the exercise intervention in the “Adherence and fidelity” section (page 12).

Line 47. Could some more detail be provided on what the functional exercises will consist of?

Response: We agree some more detail could have been provided for better clarity and insight into the training program. We have now elaborated this information on page 9.

Line 59. Re. “These organizations are encouraged to align their activities with the training principles of the RESTART trial.” – can some detail be provided on how this encouragement will be done and monitored over time? I.e. will the organisations receive training from the RESTART trial group?

Response: Yes, these organizations will indeed receive education and training. We have adjusted this sentence accordingly (page 10) as well as expanded the “Adherence and fidelity” section (page 12) to provide information on how this will be monitored.

Page 11. Line 49. Can more detail be provided on the etool? Is this a smartphone application for example? And how has this been developed?

Response: We agree that more information regarding the development, purpose, content and intended targets of the eTool would strengthen the manuscript. More information has now been added to the section describing the eTool (page 10-11).

Page 12. Lines 34 onwards. The authors should be commended for detailing how they intend to avoid, monitor and report adverse events. In line with recent guidance, I suggest they consult and adhere to the CONSORT

Harms guidance.
Junqueira DR, Zorzela L, Golder S, Loke Y, Gagnier JJ, Julious SA, et al. CONSORT Harms 2022 statement, explanation, and elaboration: updated guideline for the reporting of harms in 1090 randomised trials. BMJ. 2023 Apr 24;381:e073725

Response: Again, thank you for the positive feedback. Per the reviewer's request, we have expanded the "Harms" section on page 20-21 where details on adverse events monitoring should be described. For this we consulted the provided CONSORT Harms guidance citation, and re-checked the Harms section in the SPIRIT 2013 explanation and elaboration: guidance for protocols of clinical trials (citation below).

(Chan AW, Tetzlaff JM, Gøtzsche PC, Altman DG, Mann H, Berlin JA, Dickersin K, Hróbjartsson A, Schulz KF, Parulekar WR, Krleza-Jeric K, Laupacis A, Moher D. SPIRIT 2013 explanation and elaboration: guidance for protocols of clinical trials. BMJ. 2013 Jan 8;346:e7586. doi: 10.1136/bmj.e7586. PMID: 23303884; PMCID: PMC3541470.)

Page 12. Line 55-56. Could the authors please expand on how they intend to assess/ monitor the fidelity of the intervention? Which markers will be used to assess fidelity for example (i.e., heart rate) and will this also include researcher observations/ checklists etc?

Response: Information on this has now been added to the Adherence and fidelity section on page 12.

Page 16. Line 53. Please consider changing 'objective physical activity' to device-measured physical activity, here and throughout the manuscript. This is to acknowledge that a degree of subjectivity still exists when using devices to measure physical activity (e.g., which cut points are applied, epoch length etc), and the measurement is therefore not truly objective. Please also add detail on how the physical activity data will be analysed.

Response: We agree with the reviewer's insightful comment. We have now changed the phrasing accordingly throughout the manuscript to reflect that we include device-measured physical activity in the trial. We have additionally added some information regarding the analyses, where we will use a data-driven approach due to the lack of international standards and consensus regarding cut-point and epoch lengths (page 17).
